# Effect of oral glucose tolerance test-based insulin resistance on embryo quality in women with/without polycystic ovary syndrome

**DOI:** 10.3389/fendo.2024.1413068

**Published:** 2024-06-24

**Authors:** Zhengyan Hu, Rujun Zeng, Yuanting Tang, Yingjun Liao, Tao Li, Lang Qin

**Affiliations:** ^1^ The Reproductive Medical Center, Department of Obstetrics and Gynecology, West China Second University Hospital, Sichuan University, Chengdu, Sichuan, China; ^2^ Key Laboratory of Birth Defects and Related Diseases of Women and Children (Sichuan University), Ministry of Education, Chengdu, Sichuan, China; ^3^ West China School of Medicine, Sichuan University, Chengdu, Sichuan, China; ^4^ Department of Laboratory Medicine, West China Second University Hospital, Sichuan University, Chengdu, China; ^5^ Department of Outpatient, West China Second University Hospital, Sichuan University, Chengdu, China; ^6^ Department of Obstetrics and Gynecology, West China Second University Hospital, Sichuan University, Chengdu, China

**Keywords:** polycystic ovary syndrome, insulin resistance, oral glucose tolerance test, embryo morphological assessment, *in vitro* fertilization

## Abstract

**Objective:**

To explore the effects of insulin resistance (IR) on embryo quality and pregnancy outcomes in women with or without polycystic ovary syndrome (PCOS) undergoing *in vitro* fertilization (IVF)/intracytoplasmic sperm injection (ICSI).

**Methods:**

A retrospective cohort study concerning patients with/without PCOS who received gonadotropin-releasing hormone (GnRH)-antagonist protocol for IVF/ICSI from January 2019 to July 2022 was conducted. All the patients included underwent oral glucose tolerance test plus the assessment of insulin release within 6 months before the controlled ovarian stimulation. The Matsuda Index was calculated to diagnose IR. Two populations (PCOS and non-PCOS) were included and each was divided into IR and non-IR groups and analyzed respectively. The primary outcome was the high-quality day 3 embryo rate.

**Results:**

A total of 895 patients were included (751 with PCOS and 144 without PCOS). For patients with PCOS, the IR group had a lower high-quality day 3 embryo rate (36.8% vs. 39.7%, p=0.005) and available day 3 embryo rate (67.2% vs. 70.6%, p<0.001). For patients without PCOS, there was no significant difference between the IR and non-IR groups in high-quality day 3 embryo rate (p=0.414) and available day 3 embryo rate (p=0.560). There was no significant difference in blastocyst outcomes and pregnancy outcomes for both populations.

**Conclusion:**

Based on the diagnosis by the Matsuda Index, IR may adversely affect the day 3 embryo quality in patients with PCOS but not pregnancy outcomes. In women without PCOS, IR alone seems to have less significant adverse effects on embryo quality than in patients with PCOS. Better-designed studies are still needed to compare the differences statistically between PCOS and non-PCOS populations.

## 1 Introduction

Insulin resistance (IR) refers to a state in which target tissues are less responsive to physiological insulin levels, related to acquired conditions and genetic factors (1, 2). At the molecular level, it can be caused by any disruption in insulin signaling pathways (3). IR impairs metabolic processes and is thought to be associated with the development of many modern diseases, including obesity, type 2 diabetes, cardiovascular diseases, and fatty liver (1). The overall prevalence of IR might be over 40%, however, it is still considered underestimated (4, 5).

IR, as a key pathophysiological feature of polycystic ovary syndrome (PCOS), has been found in more than 60% of women with PCOS (6, 7). PCOS is now considered an endocrine disorder characterized by hyperandrogenism, ovulatory dysfunction, and polycystic ovary morphology, affecting a great number of reproductive-aged women (8, 9). Since PCOS is often associated with anovulatory infertility, interventions are needed for some patients with PCOS who want to achieve pregnancy. Usually, *in vitro* fertilization (IVF) is considered when lifestyle improvements and ovulation induction do not result in a successful pregnancy (10). Previous studies have shown that patients with PCOS can achieve a satisfactory live birth rate with IVF, but they still face a higher risk of adverse outcomes during IVF and pregnancy maintenance, including lower fertilization and miscarriage (11–14). It has been questioned that glucose regulation and insulin homeostasis may be related to the less ideal reproductive outcomes for patients with PCOS by affecting both the embryos and their developmental environment (15). Besides, some studies have shown that adverse reproductive outcomes due to insulin disorders may also exist independently of PCOS (16).

So far, a few studies have already focused on the impact of IR on IVF outcomes (16–21). However, their methods of assessing IR are not uniform and their conclusions are not agreed upon. There are also few studies comparing the impact of IR with and without the presence of PCOS (21). At present, hyperinsulinemic euglycemic clamp (HEC) is the gold standard for diagnosing IR, but its complexity limits its application (5). The homeostasis model assessment of IR (HOMA-IR) is now widely used to evaluate IR in clinical research. However, assessment based only on fasting status limits its sensitivity (22). In this retrospective study, IR was assessed based on the oral glucose tolerance test (OGTT) with the assessment of insulin release. The Matsuda Index was calculated to recognize IR. We focused on patients with PCOS and also included patients without PCOS, intending to explore the impact of IR on embryo quality and pregnancy outcomes among women with/without PCOS undergoing IVF/intracytoplasmic sperm injection (ICSI) -embryo transfer (ET) treatment.

## 2 Materials and methods

### 2.1 Study design

We conducted a single-center retrospective cohort study at West China Second University Hospital, Sichuan University. Patients who received gonadotropin-releasing hormone (GnRH)-antagonist protocol for their first IVF/ICSI cycle from January 2019 to July 2022 were included. This study was approved by the Ethics Committee of West China Second University Hospital and written informed consent was waived.

Infertility is defined as the failure to be pregnant after at least 12 months of regular unprotected sexual intercourse. All the patients receiving their first IVF/ICSI cycle with GnRH-antagonist protocol for controlled ovarian stimulation (COS) due to primary or secondary infertility were screened in the electronic medical record management system. PCOS was diagnosed according to the Rotterdam criteria, which required at least two of the following three criteria: oligo/amenorrhea, clinical or biochemical hyperandrogenism (modified Ferriman-Gallwey score ≥5 and/or total testosterone (T) ≥0.40ng/ml) and polycystic ovary morphology (2–9mm antral follicle count (AFC) ≥20 or ovarian volume ≥10 cm^3^ on ultrasonography), with other causes of hyperandrogenism and ovulation dysfunction excluded. A group of patients without PCOS were also included. These patients have a normal ovarian function, with two ovaries, a follicle-stimulating hormone (FSH) of >0 and ≤10 IU/L, an AFC of >5 and ≤15, an anti-mullerian hormone (AMH) of ≥1.4 and ≤5 ng/mL, and a regular menstrual cycle. They underwent IVF/ICSI due to definite pelvic and/or tubal infertility. All the patients included underwent OGTT plus insulin release test within 6 months before the COS.

Exclusion criteria included: 1) with other endocrine diseases (such as thyroid diseases and diabetes mellitus) or immune diseases (such as systemic lupus erythematosus and antiphospholipid syndrome); 2) with chromosomal abnormalities; 3) cycles with preimplantation genetic testing; 4) without complete clinical information.

### 2.2 OGTT, Matsuda Index, and IR

OGTT with the insulin release test was performed within 6 months before the COS. Patients were asked to ingest a solution containing 75g of glucose after an 8-hour fast, and blood samples were collected at 0, 0.5, 1, and 2 hours to determine blood glucose and insulin. Impaired glucose tolerance (with or without impaired fasting glucose) was defined as fasting blood glucose <7.0mmol/L and 2-hour blood glucose ≥7.8mmol/L and <11.1mmol/L. The four-point Matsuda Index was used to evaluate IR, which was calculated as 10,000/√[fasting glucose (mg/dL) × fasting insulin (μU/mL) × mean glucose (mg/dL) × mean insulin (μU/mL) during OGTT]. The conversions are 1μU/mL = 6pmol/L for insulin and 1mmol/L = 18mg/dL for glucose. Mean glucose and insulin were calculated by the trapezoid method (23, 24). IR was determined if the Matsuda Index was less than 4.2 (24).

### 2.3 COS and IVF/ICSI-ET

All the patients received the GnRH antagonist protocol. COS was started on day 2 of the menstrual cycle with one of the following gonadotropin (Gn) preparations: recombinant FSH (GONAL-F, Merck Serono, Italy; or Puregon, Organon, The Netherlands), urinary FSH (Lizhu Pharmaceutical Trading Co., China) or highly-purified hMG (Menopur, Ferring, Germany) preparations. The starting dose was 150–300 IU/day and the daily dose remained unchanged unless the serum estradiol (E2) did not increase after 7 days. The pituitary gonadotrophin suppression was started with a GnRH antagonist (Injection Cetrotide acetate, Aeterna Zentaris, Canada) at a dose of 0.25mg/d on the day 6 of COS, or the day the dominant follicle reached 14mm diameter or serum E2 reached 300pg/ml. As soon as two follicles ≥ 18mm or three follicles ≥ 17mm diameter were detected, ovulation trigger was performed by human chorionic gonadotropin (hCG; Lizhu Pharmaceutical Trading Co., China) at a dose of 8000–10000 depending on the patient’s weight IU or recombinant hCG (rhCG; Lizhu Pharmaceutical Trading Co., China) at a dose of 250μg. For patients with a high risk of OHSS, 4000–5000 IU of hCG, 0.2mg of GnRH agonist (Diphereline, Ipsen, Ferring, Germany), or 2000IU of hCG plus 0.2mg of GnRH agonist was used. Oocytes were retrieved by transvaginal ultrasound-guided aspiration 36–38h after the trigger. IVF/ICSI was performed depending on the medical history. Ultrasound-guided fresh ET was conducted on day 3 or day 5 after oocyte retrieval. All patients received luteal phase support with intramuscular progesterone (60 mg per day) or vaginal progesterone gel (90 mg per day) combined with oral dydrogesterone (20 mg per day). Implantation was assessed by measurement of serum β-hCG concentrations 2 weeks after ET and pregnancy was confirmed by transvaginal ultrasound 3–4 weeks after that.

### 2.4 Embryo morphological assessment

Oocyte maturity was assessed soon after the oocyte retrieval (day 0), and fertilization was assessed on day 1. Embryo morphological assessment was performed daily, and high-quality embryos and available embryos were identified on day 3 and day 5. Day 3 (D3) embryos were categorized into grades I, IIa, IIb, IIIa, IIIb, and IV according to the number of cells and degree of fragmentation, from best to worst (25). Embryos of grade I and IIa were regarded as high-quality and embryos of grade I, II, and IIIa were regarded as available. Day 5 embryos (blastocysts) were graded based on the quality of trophectoderm (A, B, C) and inner cell mass (A, B, C) (26). Embryos of grades AA, AB, BA, and BB were regarded as high-quality and all except for grade CC were regarded as available.

### 2.5 Outcomes

Demographic and medical history characteristics including age, body mass index (BMI), duration of infertility, type of infertility, AMH, baseline sex hormone, and AFC were collected from the electronic medical record management system. Outcomes of the COS included starting, daily, and total dose of Gn, sex hormones and endometrium thickness on the trigger day, number of oocytes retrieved, number of metaphase II (MII) oocytes, fresh ET cancellation rate, and severe OHSS rate. The measurements of all sex hormones were performed in the same laboratory using competitive chemiluminescent immunoassay (CLIA, Siemens ADVIA CENTAUR), and the normal ranges are shown in **Supplementary Table S1**. Outcomes of embryo morphological assessment included percentage of each D3 embryo grade, high-quality and available D3 embryo rate (per normally fertilized oocyte), and high-quality and available blastocyst rate (per formed blastocyst). Pregnancy outcomes included implantation rate (per ET), clinical pregnancy rate (per ET), miscarriage rate (per clinical pregnancy), and live birth rate (per ET).

The primary outcome was the high-quality D3 embryo rate. The secondary outcomes included D3 embryo morphological grade, available D3 embryo rate, live birth rate, implantation rate, clinical pregnancy rate, and miscarriage rate.

### 2.6 Statistical analysis

Two populations (PCOS and non-PCOS) were included and each population was divided into two groups (IR group and non-IR group). The two populations were statistically analyzed respectively. A Kolmogorov-Smirnov test was used to estimate the normality of distribution for continuous variables. Normally distributed variables were presented as mean ± standard deviation (SD) and analyzed by Student’s t-test. Non-normally distributed variables were presented as median (25^th^-75^th^ percentiles) and analyzed by Mann-Whitney U test. Categorical variables were presented as number of cases (percentage) and analyzed by chi-square or Fisher’s exact test as appropriate. The adjusted differences of the high-quality D3 embryo rate and the pregnancy outcomes were further analyzed by binary logistic regression analysis and presented as odds ratio (OR), 95% confidence intervals (CI). P-value of less than 0.05 was regarded as statistically significant. All analyses were performed using the SPSS version 26.0 (SPSS Inc., Chicago, IL, UPL).

## 3 Results

### 3.1 Baseline characteristics and endocrine profiles

A total of 895 patients were included in this retrospective cohort study, with 751 patients in the PCOS groups (340 IR and 411 non-IR) and 144 patients in the non-PCOS groups (104 IR and 40 non-IR). As shown in **Table 1**, for patients with PCOS, BMI was higher in the IR groups (23.80 ± 3.08 vs. 22.11 ± 3.11, p<0.001), AMH was lower in the IR group (p=0.004) and baseline T was statistically higher in the IR group (p=0.003). The fasting glucose, 2-hour glucose, fasting insulin, and 2-hour insulin were statistically higher in the IR group (p<0.001). The impaired glucose tolerance was comparable. The median of Matsuda index was 2.23 in the IR group and 6.51 in the non-IR group. For patients without PCOS, BMI was higher in the IR groups (23.23 ± 3.11 vs. 21.39 ± 2.19, p<0.001), and age and type of infertility were significantly different (p<0.05). The fasting glucose, 2-hour glucose, fasting insulin, and 2-hour insulin were statistically higher in the IR group (p<0.005). The impaired glucose tolerance was comparable. The median of Matsuda index was 2.71 in the IR group and 5.52 in the non-IR group.

**Table 1:** Baseline characteristics.

	PCOS (N=751)			non-PCOS (N=144)		
	IR (n=340)	non-IR (n=411)	P-value	IR (n=104)	non-IR (n=40)	P-value
Age (y)	29.8 ± 3.6	29.3 ± 3.5	0.104	30.3 ± 2.9	31.3 ± 2.0	0.017
BMI (kg/m^2^)	23.80 ± 3.08	22.11 ± 3.11	<0.001	23.23 ± 3.11	21.39 ± 2.19	<0.001
Duration of infertility (y)	3.7 ± 2.5	3.2 ± 2.3	0.001	3.5 ± 2.6	2.6 ± 2.0	0.053
Type of infertility [n (%)]			0.151			0.021
Primary infertility	225 (66.2)	292 (71.0)		71 (68.3)	19 (47.5)	
Secondary infertility	115 (33.8)	119 (33.8)		33 (31.7)	21 (52.5)	
AMH (ng/mL)	9.38 ± 4.75	10.40 ± 4.76	0.004	2.84 ± 1.03	2.81 ± 1.03	0.373
OGTT						
Fasting glucose (mmol/L)	5.13 ± 0.55	4.88 ± 0.35	<0.001	5.10 ± 0.43	4.89 ± 0.32	0.004
2-hour glucose (mmol/L)	7.53 ± 2.06	6.06 ± 1.60	<0.001	7.52 ± 1.80	6.46 ± 1.80	0.001
Fasting insulin (pmol/L)	89.21 ± 49.56	44.25 ± 20.37	<0.001	82.08 ± 55.45	39.74 ± 14.27	<0.001
2-hour insulin (pmol/L)	718.87 ± 370.64	318.15 ± 208.51	<0.001	635.38 ± 341.87	278.76 ± 107.55	<0.001
Matsuda index	2.23 (1.56–3.05)	6.51 (4.91–8.71)	<0.001	2.71 (1.92–3.21)	5.52 (4.61–6.58)	<0.001
Impaired glucose tolerance [n (%)]	78 (22.9)	79 (19.2)	0.212	39 (37.5)	11 (27.5)	0.259
Baseline sex hormone						
FSH (IU/L)	6.40 (5.40–7.60)	6.40 (5.40–7.60)	0.359	7.25 (6.20–8.20)	7.25 (6.30–8.10)	0.700
LH (IU/L)	7.90 (5.10–11.82)	8.10 (4.90–12.40)	0.879	4.20 (2.92–6.12)	4.53 (2.92–6.97)	0.603
T (ng/dL)	40.0 (30.0–51.0)	36.0 (27.0–48.0)	0.003	25.0 (20.0–30.0)	24.0 (20.0–34.8)	0.948
LH/FSH	1.30 (0.84–1.91)	1.27 (0.80–1.95)	0.487	0.58 (0.43–0.90)	0.66 (0.38–0.95)	0.457
AFC	20.1 ± 7.0	20.1 ± 6.6	0.959	9.9 ± 2.6	9.7 ± 2.7	0.216

Data are presented as mean ± SD, median (25th-75th percentiles), or number (percentage).

PCOS, polycystic ovary syndrome; IR, insulin resistance; BMI, Body Mass Index; AMH, anti-mullerian hormone, 1ng/mL=7.14pmol/L; OGTT, oral glucose tolerance tests; LH, luteinizing hormone; T, testosterone, 1ng/dl=0.0347nmol/L; AFC, antral follicle count.

### 3.2 Effects of IR on controlled ovarian stimulation

As shown in **Table 2**, for patients with PCOS, IR group had longer days of Gn use (p=0.028), a higher total Gn dosage (p<0.001), a lower trigger day E2 (p=0.029), a higher trigger day luteinizing hormone (LH) (p=0.015), and a lower fresh ET cancellation rate (p=0.035). The number of oocytes retrieved, the number of MII oocytes, MII oocyte rate, and severe OHSS rate were comparable between the IR and non-IR groups. For patients without PCOS, the IR group had statistically more oocytes retrieved (p=0.021) and more MII oocytes (p=0.010). The duration of Gn use, total Gn dosage, trigger day indicators, and fresh ET cancellation rate were comparable between the IR and non-IR groups. No severe OHSS was reported in patients without PCOS. The MII oocyte rate was comparable between the IR group and non-IR group for all patients.

**Table 2:** Outcomes of the controlled ovarian stimulation.

	PCOS (N=751)			non-PCOS (N=144)		
	IR (n=340)	non-IR (n=411)	P-value	IR (n=104)	non-IR (n=40)	P-value
Duration of Gn use (d)	10.0 ± 1.8	9.7 ± 1.4	0.028	10.1 ± 1.3	9.9 ± 1.7	0.450
Total Gn dose (IU)	1957.70 ± 759.71	1720.10 ± 571.17	<0.001	2511.30 ± 662.12	2437.50 ± 634.25	0.546
Trigger day						
E2 (pg/mL)	4412.80 (2842.70–6552.00)	4987.55 (3021.62–7370.50)	0.029	2633.90 (1669.60–3429.05)	2145.95 (1603.32–2901.40)	0.152
P (ng/mL)	1.01 (0.76–1.39)	1.02 (0.72–1.36)	0.429	0.94 (0.66–1.23)	0.85 (0.62–1.12)	0.187
LH (IU/L)	2.40 (1.40–4.10)	2.00 (1.20–3.60)	0.015	1.80 (1.10–2.70)	1.80 (1.30–2.88)	0.328
Em thickness (mm)	5.29 ± 1.08	5.25 ± 0.98	0.571	5.36 ± 1.04	5.18 ± 1.12	0.369
No. of oocytes retrieved	16.7 ± 7.6	16.4 ± 8.2	0.543	10.1 ± 5.0	8.3 ± 3.6	0.021
No. of MII oocytes	14.1 ± 7.1	13.7 ± 7.7	0.835	8.4 ± 4.5	6.7 ± 3.1	0.010
MII oocyte rate [n(%)]	4809/5694 (84.5)	5634/6737 (83.6)	0.209	876/1047 (83.7)	267/332 (80.4)	0.171
Fresh ET cancellation rate [n(%)]	224 (65.9)	300 (73.0)	0.035	46 (44.2)	16 (40.0)	0.646
Severe OHSS rate [n(%)]	7 (2.1)	12 (2.9)	0.455	/	/	/

Data are presented as mean ± SD, median (25th-75th percentiles), or number (percentage).

Gn, gonadotropin; GnRH, gonadotropin releasing hormone; LH, luteinizing hormone; E2, estradiol, 1pg/mL=3.67pmol/L; P, progesterone, 1ng/mL=3.18nmol/L; Em, endometrium; MII, metaphase II oocytes; OHSS, ovarian hyperstimulation syndrome. /, No cases of severe OHSS have been reported in patients without PCOS.

### 3.3 Effects of IR on embryo morphological assessment

As shown in **Table 3**, the ART methods were comparable between the IR and non-IR groups in both populations, and there was no significant difference in the high-quality and available blastocysts rate. For patients with PCOS, the IR group had a significantly lower high-quality D3 embryo rate (36.8% vs. 39.7%, p=0.005) and available D3 embryo rate (67.2% vs. 70.6%, p<0.001). Specifically, the IR group had a lower percentage of grade I (p=0.028) and grade IIa D3 embryos (p=0.009), and a higher percentage of grade IIIb (p=0.008) and grade IV D3 embryos (p=0.044). For patients without PCOS, there was no significant difference between the IR and non-IR groups in embryo morphological assessment.

**Table 3:** Outcomes of ART and embryo morphological assessment.

	PCOS (N=751)			non-PCOS (N=144)		
	IR (n=340)	non-IR (n=411)	P-value	IR (n=104)	non-IR (n=40)	P-value
ART method [n(%)]			0.482			0.584
IVF	279 (82.1)	350 (85.2)		89 (85.6)	34 (85.0)	
ICSI	22 (5.4)	20 (5.9)		11 (10.6)	3 (7.5)	
IVF+ICSI	39 (9.5)	41 (12.1)		4 (3.8)	3 (7.5)	
D3 embryo morphological grade [n(%)]						
I	5/4061 (0.1)	17/4752 (0.4)	0.028	4/800 (0.5)	2/233 (0.9)	0.526
IIa	1489/4061 (36.7)	1871/4752 (39.4)	0.009	285/800 (35.6)	89/233 (38.2)	0.472
IIb	1110/4061 (27.3)	1302/4752 (27.4)	0.945	222/800 (27.8)	65/233 (27.9)	0.965
IIIa	124/4061 (3.1)	166/4752 (3.5)	0.249	36/800 (4.5)	8/233 (3.4)	0.478
IIIb	429/4061 (10.6)	422/4752 (8.9)	0.008	52/800 (6.5)	14/233 (6.0)	0.787
IV	904/4061 (22.3)	974/4752 (20.5)	0.044	201/800 (25.1)	55/233 (23.6)	0.636
High-quality D3 embryo rate [n(%)]	1494/4061 (36.8)	1888/4752 (39.7)	0.005	289/800 (36.1)	91/233 (39.1)	0.414
Available D3 embryo rate [n(%)]	2728/4061 (67.2)	3356/4752 (70.6)	<0.001	547/800 (68.4)	164/233 (70.4)	0.560
High-quality blastocysts rate [n(%)]	474/1684 (28.1)	649/2098 (30.9)	0.062	128/281 (45.6)	33/74 (44.6)	0.883
Available blastocysts rate [n(%)]	1499/1684 (89.0)	1856/2098 (88.5)	0.596	254/281 (90.4)	71/74 (95.9)	0.126

Data are presented as number (percentage).

ART, assisted reproductive technology; IVF, in vitro fertilization; ICSI, intracytoplasmic sperm injection; D3, Day 3.

As shown in **Table 4**, after adjusting for age, BMI, baseline T, duration of infertility, and AMH, the high-quality D3 embryo rate was still statistically lower in the IR group for patients with PCOS (adjusted OR: 0.893, 95% CI: 0.816–0.978, p=0.015), and there was no significant difference between the two groups for patients without PCOS (p=0.270). In multivariable regression, IR (adjusted OR: 0.897, 95% CI: 0.820–0.982, p=0.018) and higher BMI (adjusted OR: 0.985, 95% CI: 0.971–1.000, p=0.048) were found to affect D3 embryo quality in PCOS patients significantly.

**Table 4:** Adjusted outcomes for patients with/without PCOS.

	PCOS			non-PCOS		
	Adjusted OR (95% CI)		P value	Adjusted OR (95% CI)		P value
High-quality D3 embryo rate	IR	0.893 (0.816–0.978)	0.015	IR	0.829 (0.594–1.157)	0.270
Multivariable regression	IR	0.897 (0.820–0.982)	0.018			
	BMI	0.985 (0.971–1.000)	0.048			
Clinical pregnancy rate	IR	0.819 (0.467–1.435)	0.485	IR	0.857 (0.265–0.265)	0.797
Multivariable regression	Age	0.928 (0.864–0.996)	0.040			
Miscarriage rate	IR	0.749 (0.266–2.108)	0.584	IR	0.196 (0.008–4.710)	0.315
Multivariable regression	AMH	1.137 (1.024–1.264)	0.017			
Live birth rate	IR	0.854 (0.486–1.499)	0.581	IR	1.250 (0.352–4.432)	0.730

Adjusting for age, body mass index (BMI), testosterone, duration of infertility, and anti-mullerian hormone (AMH). Non-IR group= “0”, IR group= “1”. P<0.05 was regarded as statistically different.

### 3.4 Effects of IR on pregnancy outcomes

As shown in **Table 5**, there was no significant difference in the implantation rate, clinical pregnancy rate, miscarriage rate, and live birth rate between the IR and non-IR groups for all patients.

**Table 5:** Pregnancy outcomes.

	PCOS (N=751)			non-PCOS (N=144)		
	IR (n=340)	non-IR (n=411)	P-value	IR (n=104)	non-IR (n=40)	P-value
Implantation rate [n(%)]	67/116 (57.8)	66/111 (59.5)	0.795	27/58 (46.6)	10/24 (41.7)	0.686
Clinical pregnancy rate [n(%)]	59/116 (50.9)	60/111 (54.1)	0.630	20/58 (34.5)	7/24 (29.2)	0.641
Miscarriage rate [n(%)]	8/59 (13.6)	13/60 (21.7)	0.246	3/20 (15.0)	2/7 (28.6)	0.426
Live birth rate [n(%)]	50/116 (43.1)	47/111 (42.3)	0.908	17/58 (29.3)	5/24 (20.8)	0.431

Data are presented as number (percentage).

As shown in **Table 4**, after adjusting for age, BMI, baseline T, duration of infertility, and AMH, there was still no significant difference in the clinical pregnancy rate, miscarriage rate, and live birth rate between the two groups for all patients. In multivariable regression, age was found to significantly affect clinical pregnancy rate (adjusted OR: 0.928, 95% CI: 0.864–0.996, p=0.040) and AMH was found to significantly influence miscarriage rate (adjusted OR: 1.137, 95% CI: 1.024–1.264, p=0.017) in PCOS patients.

## 4 Discussion

This is a single-center retrospective cohort focused on the influence of IR on embryo quality and pregnancy outcomes in both patients with and without PCOS. In this study, we found that IR may have adverse effects on the embryo morphological grading of the D3 embryos in patients with PCOS but not blastocyst quality or pregnancy outcomes. In women without PCOS, we did not find significant effects of IR on embryo quality and pregnancy outcomes.

### 4.1 The pathophysiological changes of IR

The insulin-AKT signaling network, consisting of elements such as the insulin receptor, insulin receptor substrate, phosphatidylinositol 3-kinase, AKT, and AKT substrates, controls metabolism (27). IR with some of its effects is assumed to be due to a defect in the pathways (27). As it has been observed, there are several molecular interactions between IR and obesity (28), manifested as higher BMI in people with IR (17, 29). As for reproductive health, the insulin-like growth factor (IGF) family, including insulin, IGF-1, and IGF-2, are of great importance in regulating reproductive development and function (30). In the physiological state, insulin may play a role in folliculogenesis by promoting oocyte growth, hormone synthesis, and cell proliferation of granulosa and theca cells, including androgen production in theca-interstitial cells (31). In the pathology of IR, the androgen production of ovaries and adrenal glands was enhanced, leading to elevated local and circulating androgen (32). Hyperandrogenism may also contribute to metabolic dysfunction in turn (32–35). Besides, although PCOS is now thought to be associated with a variety of complex environmental and genetic factors, hyperandrogenism is regarded as the main cause of PCOS (36, 37). It is noteworthy that with the existence of PCOS, the vicious cycle between IR and hyperandrogenism seems to be stronger (37).

### 4.2 Effects of IR on the controlled ovarian stimulation

The effects of IR on the COS have been noticed recently. According to previous studies (29, 38) and our results, during the controlled ovarian stimulation, patients with PCOS accompanied by IR had a higher total Gn dosage applied, but lower E2 levels on the trigger day. On the one hand, it may be because patients with IR tend to have higher BMI. On the other hand, it suggests that IR may affect ovarian sensitivity to FSH preparations in patients with PCOS, which may also explain the significantly lower fresh ET cycle cancellation rate. Some studies also showed a tendency for the number of oocytes retrieved to decrease with increasing IR levels in patients with PCOS (20, 29, 38), while our results did not, which may be related to differences in how IR was diagnosed. Several pathways discovered only in patients/models with PCOS concerning FSH receptors and insulin/IGF have been researched, which may help to explain the phenomenon that the ovarian response to FSH preparations tends to decrease with the increasing IR degree only in patients with PCOS (31, 39, 40). However, the mechanism by which IR influences ovarian sensitivity in patients with PCOS remains unclear.

Unexpectedly, in patients without PCOS, with higher BMI, similar AMH, and similar Gn dosage, the IR group retrieved more oocytes than the non-IR group. In previous studies, Wang et al. reported numerically more oocytes retrieved in the IR group (16). Yang et al. reported similar oocytes retrieved between the non-IR group and mild IR group and fewer oocytes retrieved in the severe IR group, though the numerical differences are very small (21). Whether mild IR in non-PCOS patients improves ovarian response might be worth exploring further.

### 4.3 Effects of IR on embryo quality

Although IR is thought to have negative effects on oocyte and subsequent embryo quality, results from clinical trials were controversial (41–44). As for the oocyte quality, in our study, the oocyte maturation rate was comparable between the IR and non-IR groups in both populations. For patients without PCOS, our findings are contrary to Wang et al (16). And for patients with PCOS, our result was consistent with previous studies (17, 21). However, it is noteworthy that apart from the maturation rate, oocyte size may also be an important marker for quality evaluation (41), which has not been counted in these studies.

As for the D3 embryo quality, we found that IR adversely affects D3 embryo morphological assessment in patients with PCOS. This effect was characterized by a decrease in high-quality and available embryos and an increase in poor-quality (IIIb and IV grade) embryos, which was not observed in patients without PCOS. This phenomenon might be due to the more severe metabolic disorders in patients with PCOS. Excessive insulin and androgen may impair the quality of oocytes and induce metabolic disorders in theca cells and granulosa cells (45, 46). When PCOS exists, the situation becomes more complicated due to the vicious cycle between hypothalamic-pituitary-ovarian (HPO) axis disorders and metabolic disorders (47). However, considering several numerical differences in the embryonic outcomes between the IR and non-IR groups in the non-PCOS population, which is similar to the PCOS population, the different results between the two populations may be due to sample size and statistical power. Besides, previous clinical studies (17, 21, 48) did not show similar outcomes of high-quality or available D3 embryo rate in patients with PCOS, which may be related to the diagnosis of IR and the criteria for embryo morphological evaluation.

As for the blastocyst quality, we did not observe an effect of IR on the high-quality or available blastocyst rate. This may be because after the best D3 embryos have been frozen or transferred and the worst ones discarded, the remaining embryos have undergone a screening process and the difference in quality may be smaller. Besides, blastocysts were cultured in a standard embryo culture medium for a longer time, so DNA damage repair mechanisms may have played a greater role in the reparable effects of IR (49). In addition, after the cleavage-stage embryo is formed, the paternal-derived genetic material becomes involved in the regulation of embryonic development. The expression of the embryonic genome begins to replace the role of maternal-derived transcripts, and the negative influence of factors carried by the oocyte may be reduced (50).

### 4.4 Effects of IR on pregnancy outcomes

IR may affect pregnancy outcomes in several ways, including effects on endometrial functions and environment and placental function (51–55). Physiologic IR during pregnancy can ensure the supply of glucose to the fetus, but excessive IR may impair the endocrine metabolic regulation of the placenta, with adverse effects on both the mother and the fetus (52), causing adverse pregnancy outcomes.

However, the results of clinical studies are very inconsistent. In this study, we did not find any significant effects of IR on pregnancy outcomes. In previous studies, adverse effects of IR in patients with PCOS were reported on the clinical pregnancy rate by Chang et al. (17), and on early miscarriage rate and live birth rate by Chen et al. (20). In patients without PCOS, the adverse effect of IR on late miscarriage rate was reported by Yang et al. (21). To date, there is no agreement on the results of each pregnancy outcome. On the one hand, although the patients may have received different numbers of high-quality embryos, they all had only the best transferred. On the other hand, the IR status of patients may have changed due to the progression of the pregnancy and the interventions used, but the indicators of IR in the studies were measured only before COS. Therefore, the results of pregnancy outcomes should be treated cautiously.

Besides, in patients with PCOS, the miscarriage rate seemed to increase with AMH, which is controversial in previous studies (56). Future studies could focus on this issue.

### 4.5 Strength and limitation

This study included both participants with and without PCOS, which can better reflect the impact of IR apart from PCOS. In terms of IR diagnosis, the gold standard for diagnosing IR is the HEC technique, which is rarely used in clinical practice because of its complicated operation and expensive price. Most of the previous studies used the HOMA-IR as the criterion for diagnosis, which evaluates IR only by fasting blood glucose and insulin (5). In this study, we performed OGTT and used Matsuda Index to recognize IR. Considering that our study population is Asian, we chose 4.2 as the cut-off point of the four-point Matsuda index, according to a study conducted on Japanese subjects (24). On the one hand, compared to the HEC technique, it is easy to operate, consumes less time and money, and is therefore widely used in clinical practice. On the other hand, it takes into account insulin status in the glucose-loading state and correlates well with the gold standard (23).

There are still several limitations in our study. Firstly, this is a retrospective study and may be biased. For example, in patients without PCOS, OGTT is not a routine test and is offered only when patients have risk factors of IR/diabetes such as being overweight or smoking, or when they actively request it. As a result, there may be a bias toward patient selection. Secondly, we did not focus on indicators of pregnancy complications such as preeclampsia, gestational diabetes mellitus, and preterm birth. And we did not perform analyses of the impact of IR on pregnancy complications. Thirdly, as for pregnancy outcomes, only the outcomes of fresh ET cycles were included, and the cumulative live birth rate could not be analyzed. Fourthly, we did not correct for the severity of IR, which could have potentially influenced the results. Fifthly, we did not perform statistical comparisons between PCOS and non-PCOS patients due to large differences in sample size and biases in patient selection. And for some results, such as the embryo quality, the differences between the two groups may be due to a lack of statistical power. Therefore, these results should be treated with caution and we are committed to conducting better-designed studies in the future.

## 5 Conclusion

In conclusion, based on the diagnosis by Matsuda Index, IR may have adverse effects on the embryo morphological grading of the D3 embryos in patients with PCOS, as shown by a decrease in the percentage of high-quality embryos and an increase in the percentage of poor-quality embryos. However, IR may not impair blastocyst quality and pregnancy outcomes. For women without PCOS, IR alone seems to have less significant adverse effects on embryo quality than in patients with PCOS. Better-designed studies are still needed to compare the differences statistically between PCOS and non-PCOS populations.

## Data availability statement

The original contributions presented in the study are included in the article/**Supplementary Material**. Further inquiries can be directed to the corresponding authors.

## Ethics statement

The studies involving humans were approved by ethics committee of West China Second University Hospital. The studies were conducted in accordance with the local legislation and institutional requirements. The ethics committee/institutional review board waived the requirement of written informed consent for participation from the participants or the participants’ legal guardians/next of kin because this is a retrospective cohort study with all the identification hidden from publication.

## Author contributions

ZH: Data curation, Formal analysis, Writing – original draft. RZ: Data curation, Writing – original draft. YT: Writing – review & editing. YL: Writing – review & editing. TL: Supervision, Writing – review & editing. LQ: Conceptualization, Supervision, Writing – review & editing.
