# Supplementary material for: Effect of oral glucose tolerance test-based insulin resistance on embryo quality in women with/without polycystic ovary syndrome
Source: Front Endocrinol (Lausanne). 2024 Jun 24;15:1413068. doi: 10.3389/fendo.2024.1413068 (PMC11228294; doi:10.3389/fendo.2024.1413068)
Supplement: Supplementary file 1 [file Table_1.docx]

Supplementary Material

| **Table S1** Normal ranges of sex hormones in follicle phase | |
| --- | --- |
| Hormone | Normal range |
| E2 (pg/mL) | 19.5-144.2 |
| P (ng/mL) | 0.15-1.40 |
| LH (IU/L) | 1.9-12.5 |
| FSH (IU/L) | 2.5-10.2 |
| T (ng/dL) | 8.0-40.0 |
| E2: estradiol, 1pg/mL=3.67pmol/L; P: progesterone, 1ng/mL=3.18nmol/L; LH: luteinizing hormone; FSH: follicle-stimulating hormone; T: testosterone, 1ng/dl=0.0347nmol/L | |
